# Phosphatidylthreonine: An exclusive phospholipid regulating calcium homeostasis and virulence in a parasitic protest

**DOI:** 10.15698/mic2016.05.496

**Published:** 2016-05-02

**Authors:** Ruben D. Arroyo-Olarte, Nishith Gupta

**Affiliations:** 1 Department of Molecular Parasitology, Humboldt University, Berlin, Germany.

**Keywords:** Toxoplasma gondii, Phosphatidylthreonine, Phosphatidylserine, Optogenetics, Gene-encoded calcium indicator, Metabolically attenuated vaccine, Parasite virulence, Calcium homeostasis

Lipids are often overlooked for their functional relevance in the biological membranes, while membrane-bound proteins with their great diversity of pumps, channels and enzymes are seen as the major driving force for most dynamic processes occurring in biomembranes. Lipids are in fact much more versatile than a mere physical support for biomembranes or a rich source of energy for cells. From cholesterol, determining the assembly of integral membrane proteins in the lipid rafts, to diacylglycerol and inositides as the key secondary messengers, the diversity of lipid functions is constantly being discovered. Even more unknown is the role of those non-customary, minor lipids. One such bizarre lipid is phosphatidylthreonine (PtdThr), originally described as a byproduct of mammalian phosphatidylserine (PtdSer) synthase, when threonine was used as an alternative substrate (instead of serine) for the base-exchange reaction with phosphatidylcholine (PtdCho) or phosphatidylethanolamine (PtdEtn). In our lately published work 1, we surprisingly identified PtdThr as the major and natural phospholipid in a prevalent protozoan parasite *Toxoplasma gondii*, where it was even three to four times more abundant than the prototypical PtdSer.

To reveal the origin of this unusual phospholipid we examined the two putative base-exchange-type lipid synthases encoded by the parasite genome. When expressed in *E. coli*, both enzymes used serine as a substrate to produce PtdSer, but only one of them, henceforth called PtdThr synthase (TgPTS), was able to readily utilize threonine into PtdThr. When the *Tg*PTS gene was disrupted, not only the *de novo* synthesis of PtdThr was abolished, but also the amount of PtdSer was proportionally increased in the mutant parasites. Moreover, complementation of the mutant with an exogenous gene copy of *Tg*PTS reinstated PtdThr and reversed PtdSer to a normal level. Physiological relevance of the catalytic activity of *Tg*PTS was confirmed by expressing a *Tg*PTS isoform containing a deletion in the predicted base-exchange domain. The results also indicated that *Tg*PTS could generate PtdThr by a base-exchange mechanism similar to the one used by the mammalian PtdSer synthases to make PtdSer. *Tg*PTS and its orthologs in selected Chromalveolates (*Neospora*, *Eimeria* and *Phytophthora*) contain a series of mutations in the vicinity of the conserved substrate binding pocket when compared to the mainstream PtdSer synthases including *Tg*PSS. These residues may be critical to a divergent evolution of these two enzymes.

**Figure 1:**
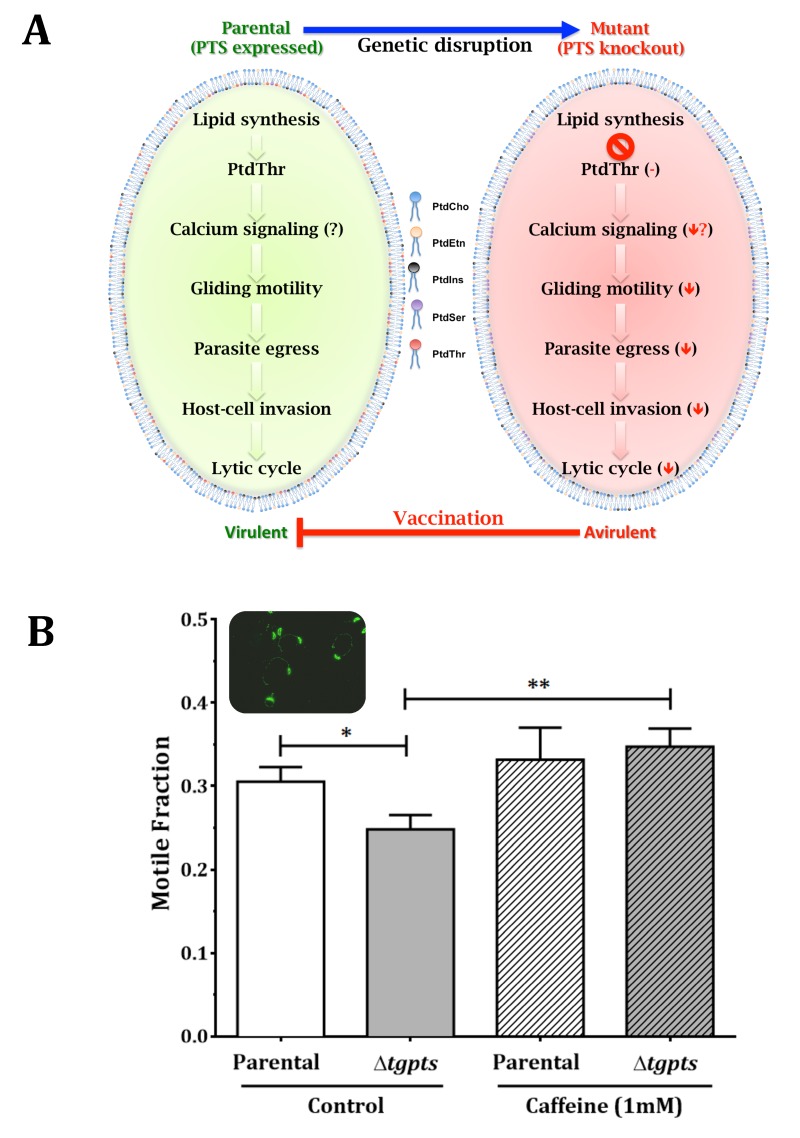
FIGURE 1: Phosphatidylthreonine-mediated regulation of calcium signaling and virulence in *T. gondii*. **(A)** Scheme illus-trating the effect of PTS gene knockout on the parasite. Image is adapted from reference 2. **(B)** Motile fraction of the indicated parasite strains treated with or without caffeine. Numbers of protein trails (immunostained with anti-Sag1 antibody) formed by extracellular parasites were counted to estimate the motile fraction.

But, what is the importance of PtdThr and can PtdSer replace its physiological function in *T. gondii*? To address these questions we resorted to a comprehensive analysis of the parasite’s lytic cycle, *i.e.* the successive steps of intracellular replication, egress and ensuing invasion of fresh host cells. Surprisingly, the loss of PtdThr in the Δ*tgpts* mutant did not affect the parasite replication, suggesting that PtdThr is dispensable for membrane biogenesis and that it can be readily replaced by its near-universal analog PtdSer in this regard. But then, why did *T. gondii* invent an enzymatic pathway to produce such a peculiar lipid? The answer lies in the other two steps of the lytic cycle, egress and invasion, both showing a noteworthy impairment in the Δ*tgpts* mutant. Egress and invasion are active processes requiring motile parasites. Therefore, parasite motility was evaluated and, as expected, it was also blunted by the lack of PtdThr and recovered upon genetic complementation with a functional enzyme. Next, we investigated whether the observed *in vitro* defects in egress, invasion and motility impacted the parasite virulence in a murine model. Animals survived the infection with the Δ*tgpts* mutant, even at 100-fold higher doses compared to control mice infected with the parental strain, which was explicitly lethal. More importantly, a single-dose infection of mice with the PTS mutant made them fully resistant to an otherwise lethal challenge with a hypervirulent strain of *T. gondii*. Moreover, the mutant-immunized mice avoided the formation of brain cysts by a cyst-forming strain and were protected against the yet-incurable chronic infection. So it appears as though *T. gondii* has evolved PtdThr during the course of evolution to optimize its parasitic lifestyle. Notably, as illustrated (Fig. 1), the pathway can be exploited to develop a metabolically attenuated vaccine.

Our study also elicited many questions, mainly regarding the lipid-mediated mechanisms regulating the lytic cycle of *T. gondii*. It has long been established that gliding motility, which underlies the observed defects in the mutant, is governed by calcium signaling. Therefore, it is likely that PtdThr influences calcium flux across the plasma membrane and/or calcium storage organelles, such as acidocalcisomes and endoplasmic reticulum. Indeed, incubation of PtdThr-deficient parasites with caffeine, a well-known agonist of ryanodine receptors/Ca_2+_ channels, restored the motile fraction to the parental level (Fig. 1B). Additional studies measuring calcium in intracellular parasites using an optogenetic sensor also suggest a role of PtdThr in calcium homeostasis 3. Prospective work on the biophysical and biochemical features of PtdThr shall reveal further mechanistic insights into the functioning of this exclusive lipid in *T. gondii*.
